# Malarial Fever

**Published:** 1886-01

**Authors:** 


### MALARIAL FEVERS.

In a very lengthy article in *Gaillard's Medical Monthly* on the malarial fevers now so common in South Western New England, by Rufus W. Griswold, M D., it is shown that, as the Dr. plainly states it, "because we really know no more as to what is the essential essence for the production of ague and fever than did our predecessors two thousand years ago, there still remains, and there will long continue to remain, opportunity for the aspirant after honors in pursuit of that will-o-the-wisp, the malarial poison."

"And, first, there is the theory of a change in the diathesis of diseases, which supposes that either the individual himself or the climatic conditions about him have undergone some unknown and undefinable modification that determined the *type* of fever that an individual may have, supposing he is to have a fever of some type It is generally credited by the profession, perhaps in all New England, that from say fifty to seventy-five years ago there has been what we call a change of diathesis in the essential fevers from a more pronounced sthenic to an asthenic form—say from inflammatory to the typhus—

over about all the region of country that comes into consideration in this paper, as well as elsewhere. And it is now assumed that there has, at this period of time, occurred such a change as has decided fevers to manifest themselves in the forms of intermittent and remittent instead of the forms of typhus and typhoid. As affording some ground of justification for this theory of change, it is to be noticed as a fact occurring over near all of Connecticut, with the appearance of ague and fever to much extent, typhus correspondingly decreasing. * * * What relation may be justly deduced out of the lessened quantity of typhoid with the increase of intermittent, has not been determined; but if it is to be assumed that the partial departure of typhoid, and the income of intermittent in its stead, is from diathetic change, and the change is in the *individual*, there will remain this curious and strange fact to be accounted for, to wit: the remarkable change in the disasethis of the human constitution rather suddenly came over a very large portion—at some points more than half the population of South-western Connecticut—eight or ten years after the same remarkable change came over half the residents of Connecticut Valley, in the middle of the state, and in still another eight or ten years the same remarkable change came to a large portion of the people of Rhode Island in a very sudden manner, and these points are all on the same degree of latitude; the character of the region is similar, the habits of the people are alike, their receptivities to disease are the same. * * The mere fact that intermittent came in, is no proof that it has occurred; it is not even an evidence of it. If it is said that it is in the surroundings of the individual, then it may be put in reply to that, that is simply an evasion of the point we are seeking for, and answers no purpose towards the solution of the matter desired; it does not explain what the change is; does not define what has been added, what taken away to produce the change; it does not state the *cause* Something has come, beyond doubt; what *is* that something? Simply to speak of change is not to explain the matter, but is to leave it garbed in the same obscurity. * * *

"The facts are, that when we have had intermittent in a dry season it has been attributed to the drought; when we have had the same thing in a wet season it has been attributed to the dampness; when we have had it all along with or after a hot summer it has been charged to that; and when we have had it with a cold summer it has been charged to that and all with the same unreasonable inference. What I wish to ask attention to in this connection is that, say in the decade from 1860 to 1870, when intermittent fever was raging with extreme violence in the New Haven section, attacking at some points in the same section more than half the population, as in Hamden, there was almost absolutely none of it in the Connecticut valley region, thirty miles beyond ; that in the next decade, from 1870 to 1880, when the inhabitants of that region were suffering after the like manner with their neighbors in the New Haven section, the people of the Thames region and of Rhode Island, thirty to forty miles east, had not been touched with malarial diseases. This is truth beyond dispute. And I assert that in the ten years from 1850 on, taken altogether, there was no such difference between the rain-fall or in temperature in the New Haven region and the Connecticut valley, nor any such difference in any other climatic condition whatever, as could originate and continue intermittent in great prevalence through all these years in one region while it was absent in the others. If the ability to generate malarial diseases resided in the climatic conditions in Southwestern Connecticut in every year from 1860 for ten years, a like ability would have been demonstrated to residents in precisely similar climatic conditions a few miles further east. and would have been developed there. I assert, further, that in the ten years from 1870 on, taking them altogether, there was no such difference between the climatic conditions in the Conneticut valley, and those in Eastern Connecticut and Rhode Island, either as to heat, cold, moisture or dryness, or in any other form, as could originate and perpetuate, in all these years a virulent assistance of malarial disease in the one section, while the other section remained wholly exempt. I make these assertions with the utmost confidence that they cannot be contradicted. The logic of the facts in this connection is irresistable ; It cannot be undermined. Going a step further in consideration of a change in climatic conditions as to the cause of ague and fever, we are justified in saying this as to the entire region over which the disease has spread in the past twenty-four years, that there was no such change of rainfall or temperature between all the decades from 1860 backwards for twenty years as would account for agues appearance and continuance in every year of the last two decades in a territory where it has been absent for the seven decades before. There was no more ability in the climatic conditions of central Connecticut after 1870 to originate miasmatic disease for every year till now, than there had been in at least some of the years for fifty previous to that time, and when none appeared. There was no more ability in the climatic condition of Rhode Island to generate ague and fever in the four years from 1880 on, than in the climatic condition for the scores of years to that time. There was no such change in the climate of the hills and valleys of Berkshire County after 1877 as would develop ague where it has almost never been known."

The Dr. then alludes to the theory advanced by a portion of the medical faculty and others, that this disease was generated from the use of manure taken from New York city and spread on a large portion of the farms of a section where the ague came ; but that theory is at once set aside by the fact that miles away, where no such influence prevailed ague is just as prevalent. He says : "Intermittent has been just as bad in sections entirely removed from any possible influence from imported manure, as in sections into which it has been carried." The charge of the change of water from wells to other sources is charged with producing the ague ; but it fails entirely as the Dr. shows most conclusively, and he shows in an equally conclusive manner that the child in the nurses arms, who is carefully excluded from the evening air, to the octogenarian unable to leave his room, all have the ague. The saying that in certain points only those residing on the ground floor have the disease is as effectually disposed of. "The simple fact that an ague is contracted in a certain locality, whether by visit or a permanent resident, is nothing whatever towards explaining what the essential essence of that ague is ; it does not reach down to the bottom—the final, the *necessary* entity of the disease."

To the statement that the turning up the earth by building of railroads, he gives a very thorough history of the railroad building in the malarial districts of Connecticut, which shows that the ague did not appear till all the railroads in that part of the state had been built from forty years to a late period, but so long that all the roads look old and time worn. No ague appeared while the roads were being built and the earth disturbed, but it came years afterwards. The Dr. says all the railroads were built twenty or twenty-five years before any ague was heard of near them. The sewers of cities are charged with being the cause of malaria in the cities, but the same disease occucurs where no sewers, gasses or other city appliances are within thirty miles. In the course of his long article on the subject, he examins every theory, everything that is attempted to account for the ague. He quotes Dr. Smart of the (U. S.) army, who says : "Malaria is a disease that knows no latitude or longitude, and prevails at any altitude." And then he says : "It is acknowledged to be an inhabitant of the arctic regions. In this country it has been found as far north as Hudson's Bay. In regions where it is at all prevalent, it is active in every month of the year. Zero weather does not kill the disease in those who have it, nor prevent its appearance in many who have never been troubled with it before.

These facts eliminate the *necessity* of heat for the production of the troubles." Professor Chadburne (late of Williams College) found it in the Rocky Mountains, "where the few springs, flowing from the melted snow, are as pure as our New England trout brooks, and most of the soil is poor in organic matter." "Vegetation," said Dr. Wm. Ferguson, in relation to the matter, "is not necessary, the peculiar poison may abound where there is no decaying vegetable matter and no vegetable matter to decay" * * * The soil at the encampment of a Bristol army, on the island of Walcheren, where 700 of the troops died in six weeks of a malarial fever, in 1807, was fine, white sand, without the presence of any vegetable decomposition whatever."

We have copied but a small part of the exhaustive article of Dr. Griswold. He does not, like most M D.'s assume to know what the real cause of ague is, and he has shown in this article that he is much wiser than the man who pretends to know. In conclusion, the Dr. says: "The conclusion is, that ultimately the so-called malarial diseases will disappear from most of those parts of New England now infected with them. Silently, but not suddenly, they will be gone, and as mysteriously as they have entered. About the apparently pestiferous marsh where, for several years there have been many cases, no amount of the most careless exposure will develop a solitary straggler. New railroads may be built, new sewers constructed, new streets laid out, ponds be made and ponds be emptied, garbage be left to rot and filth to accumulate, swamps to be drained or to be left undrained—all the same, intermittent fever will not be provoked into existence in a single case; and we shall be as ignorant of the cause of its exit as we have been of the reason of its entrance."

